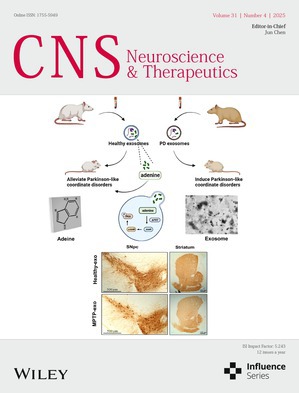# Front Cover

**DOI:** 10.1111/cns.70419

**Published:** 2025-04-28

## Abstract

The cover image is based on the article *Dual Role of Exosomes in Parkinson’s Disease: Adenine Exerts a Beneficial Effect* by Lei Chen et al., https://doi.org/10.1111/cns.70331.